# Rehabilitation challenges in multiple sclerosis

**DOI:** 10.4103/0972-2327.58273

**Published:** 2009

**Authors:** Jack S. Burks, George Kim Bigley, Harry Haydon Hill

**Affiliations:** Medicine (Neurology), University School of Medicine, Reno, Nevada; and Chief Medical Officer, Multiple Sclerosis Association of America (MSAA), Cherry Hill, New Jersey, USA; 1 Medicine (Neurology), University School of Medicine, Reno, Nevada, USA; 2 Medicine (Neurology), University School of Medicine, Reno, Nevada; and Renown Regional Center, Rehabilitation Hospital, Reno, Nevada, USA

**Keywords:** Multiple sclerosis, rehabilitation

## Abstract

While current immunomodulating drugs aim to reduce multiple sclerosis (MS) exacerbations and slow disease progression, rehabilitation aims to improve and maintain the functional abilities of patients in the face of disease progression. An increasing number of journal articles are describing the value of the many rehabilitation interventions that can be used throughout the course of the disease, from the initial symptoms to the advanced stages. An integrated team of healthcare professionals is necessary to address a myriad of problems to reduce impairments, disabilities, and handicaps. The problems may be related to fatigue, weakness, spasticity, mobility, balance, pain, cognition, mood, relationships, bowel, bladder, sexual function, swallowing, speech, transportation, employment, recreation, and activities of daily living (ADL) such as dressing, eating, bathing, and household chores. The team can help prevent complications and secondary disabilities, while increasing patient safety. Improving neurologically related function, maintaining good relationships, and feeling productive and creative adds enormously to the quality of life of people with MS and their families. Rehabilitation is more than an ‘extra’ service that is given after medical therapies; it is an integral part of the management of the diverse set of problems encountered throughout the course of the disease. An interdisciplinary team may have many members, including physicians, nurses, physical therapists, occupational therapists, speech and language pathologists, psychotherapists, social workers, recreational therapists, vocational rehabilitation therapists, patients, families, and other caregivers.

The goals of rehabilitation team therapy[1] are to maintain general health;[2] improve impairments such as spasticity and loss of strength;[3] provide training to compensate for problems such as decline in cognitive function and constipation;[4] provide adaptive equipment and devices, such as canes and wheelchairs;[5] teach skills to enhance vocational capabilities;[6] provide counseling to help adapt and cope with the changes brought on by MS;[7] educate patients and caregivers on MS and its consequences;[8] support medical therapies to increase compliance with treatment and manage side effects;[9] and provide long-term planning guidance. For example, a well-designed and coordinated exercise program can help the patient manage the activities of daily living (ADLs) by improving strength, endurance, flexibility, mobility, balance, sexual functioning, skin integrity, and safety and by decreasing fatigue, pain, depression, anxiety, and constipation. A proactive rehabilitation approach to maximize function, relationships, and employment opportunities can lead to a higher quality of life for all patients, even those who have worsening MS.

The management of MS consists of an integrated approach,[1] which aims to reduce relapses and disease progression with immunomodulating therapies;[2] to shorten relapse severity with steroids;[3] to manage symptoms and maximize function with pharmacologic and rehabilitation strategies;[4] to reduce patient stress, anxiety, and depression;[5] and to support the family and care partners on a continual basis.[1] Rehabilitation strategies are useful throughout the disease course, from the initial symptom and recovery from exacerbations through the more advanced stages of progressive illness.[2–5]

Rehabilitation in MS addresses impairments, disabilities, and handicaps:

- *Impairments* refer to the symptoms and limitations caused directly by central nervous system (CNS) damage (e.g., decreased vision, decreased strength, spasticity, tremor, etc.)
- *Disability* refers to the reduction in function in the performance of tasks (e.g., walking, bathing, etc.).
- *Handicap* refers to the reduced ability to participate in various life situations (e.g., driving, employment, etc.) and the environmental restrictions that the patient suffers.

Medical management of symptoms is combined with rehabilitation interventions for maximum stabilization or improvement of specific functions.[6] Physicians and nurses usually direct the pharmacologic management, while an integrated team of many healthcare professionals collaborate to minimize the functional impact and the effects of the disease on disability, handicaps, and quality of life. Since MS may result in a vast number of neurological problems, an interdisciplinary health care team is essential for maximizing the patient's ability to function. Each rehabilitation team member has expertise in a specific area, and the members work together to communicate and coordinate with the patient, the family, and other team members to prioritize goals and treatment regimens. Brown and Kraft[7] have published an excellent review on exercise and rehabilitation in MS.

Rehabilitation is a significant part of overall disease management, from the time of diagnosis until death. This process includes prevention, diagnosis, acute treatment, long-term treatment, community integration, and end-of-life management.[8] This coordinated continuum of comprehensive care is driven by clinical guidelines to achieve the ‘best practice’ model. The goal is to provide the right services at the right time throughout the disease process. The prevention of secondary disabilities and handicaps, as well as of medical complications, is also part of the team's responsibilities. The optimum overall health of a patient is achievable in almost any setting. Access to other services within the community is critical. For example, vocational and advocational guidance, social services, family services, legal services, and spiritual guidance have important roles to play in reducing handicaps and improving quality of life.

Maximizing quality of life is a major goal of the rehabilitation team. Physicians tend to perceive quality of life relative to the patient's physical limitations as measured by the Expanded Disability Status Scale (EDSS). On the other hand, patients more often relate quality of life to family roles, vocation, income security, emotional stability, and cognitive abilities.[9,10] The rehabilitation team recognizes the importance of integrating these different quality-of-life perceptions to impact the patient's functioning in both physical and psychosocial environments. Therefore, the team and the patient strive to maximize his/her function and independence in home and community. The healthcare team addresses patients' issues and concerns over a broad spectrum of topics [Table 1].

**Table 1:** Aspects of an education program for people with multiple sclerosis[72]

Personal Concerns
What can I expect?
Is my life in danger?
How did I get this disease?
Can I give it anyone else (contagious, genetics)?
Are there parts of me I can trust?
Medical information
What can I do to stay healthy?
What treatments are available to decrease the disease?
How do I evaluate treatments-established and claimed?
What medical resources can I take advantage of?
What are the rationales and side effects of symptomatic treatments?
What new physical skills do I need?
When do I call my health care providers for help?
Adjustment skills
What makes me wothwhile if I cannot do what I used to do?
How do I ask for help without becoming dependent?
How do I communicate my feelings and concerns to others?
Why should I work so hard when I may get worse anyway?
How do I cope with this disease over the next fifty years?
Realistic life planning
What community resources are available to me?
What kind of work can I do?
Should I have any more children?
What will I do if I become bedridden?
What will I do if I become wheelchair bound?
What will I do if I become unable to walk very far?
What will I do if I become unable to jog?
What will I do if I become nothing different from what I am now?

Rehabilitation specialists also focus on prevention. Challenges ranging from preventing falls, muscle contractures, and decubitus ulcers to preventing employment disruption are part of the rehabilitation continuum.[11]

The intensity and setting of adequate rehabilitation services often depend on the severity of the problems and the facilities available. Studies on rehabilitation in inpatients have shown variable results on impairments, but many have shown that rehabilitation services decrease disability and improve quality of life and that these improvements last up to several months.[9,12–16] Periodically repeating inpatient rehabilitation programs may prolong the initial benefits.[17]

Most MS rehabilitation is done in the home or outpatient setting. Physical therapy is the most frequent service requested, followed by occupational therapy, psychotherapy, and massage therapy; also requested are the services of social workers and speech and language therapists.[18] Sixty-six percent of MS patients have used rehabilitation services, which is proof of the perceived value of these important interventions in MS care.

Maximizing the quality of life begins with provision of access to quality healthcare followed by education on the most troubling aspects of the disease. The ability to maintain good relationships with family and friends, as well as being able to feel productive (in employment, in family roles, in voluntary work), contributes to the overall feeling of well-being. The healthcare team has a much greater role than just diagnosing illness and providing medication.

## The interdisciplinary team

The interdisciplinary team care provides both medical and functional management for the MS patient. The functional model of care focuses on disability, handicaps, quality of life, education, and psychosocial well-being. Members of the interdisciplinary team may include physicians, nurses, a physical therapist, a speech and language pathologist, an occupational therapist, a psychotherapist, social workers, a recreational therapist, a vocational therapist, a person to provide spiritual guidance, and the patient/family members/care partners.[19–23] The roles of these therapists are outlined in Table 2. Inputs from the patient, family, and care partners are essential to the success of the rehabilitation process. Care partners (caregivers) need to be educated regarding MS and must be provided psychosocial support and respite (time to themselves) to help them withstand the strain of their arduous responsibilities.

**Table 2:** Team member contributions

**Physician**	**Speech/Language Pathologist**	**Psuchologists/Psychiatrists/Other Psycho-therapists**
Medical care	Dysarthria	Affective (depression)/anxiety/personality changes
Diagnosis	Communication strategies	Congnitive impairment: retraining and adaptation
Immunomodulating therapy	Dysphasia	Psychological problems (family, work coping)
Symptomatic treatment	Cognitive dysfunction	Clinical trials - cognitive evaluations
Team leader		Psychiatric medications
		Teach straytegies to enhance
**Nurse:**	**Patient/Family members/Care partners**	Quality of Life
MS	Goals	Family and caregiver support
	Values	Team counseling
Bowel, bladder, and sexual support	Interests	
Self-care skills	Quality of Life perspective	
Maintain therapies		
Nutrition	**Occupational Therapist**	
Skin care	Upper extremity strengh	
Follow-up	Upper extremity tone	
Clinical Trial care	Upper extremity coordination	
	Activies of daily living (ADL)	
**Physical Therapists**	Adaptive equipment	**Social Workers:**
Strength	Homemaking	Resource identification
Tome	Vocational evaluation	Counseling and family support
Balance	Energy conservation,	Disposition planning
Coordination	Fatigue management	
Fatigue management	Driving evaluation	**Recreation therapy:**
Ambulation	Wheelchair evaluation	Advocational pursuits
Safty		Skill practice
Bed Mobility		Motivation
Adaptive equipment		
General conditioning		

Health promotion strategies such as exercise, nutrition, stress management, coping skills, adherence to disease-modifying therapy, and avoidance of substance misuse also contribute to the patients' medical and psychosocial well-being.

## The team approach

To enhance the rehabilitation outcome, a systematic approach to the patients' issues is followed. The steps include:

- Evaluation and problem identification
  Each team member provides inputs related to his/her area of expertise. The evaluation includes the physical, psychosocial, vocational, and advocational aspects.
- Goal setting
  Goals are identified and prioritized. Solutions and realistic expectations are formulated. The patient's values and priorities are integrated into the process. The goals should be as specific as reasonable, and time frames to reach the goals should be estimated. Larger, complex goals are broken down into smaller, more manageable and more measureable goals. For example, ‘improving gait’ is a complex issue but it can be broken up into several smaller goals – such as ‘walking a specific distance’ – that are easier to measure and attain. Once smaller goals are attained, the motivation increases to accomplish more complex goals.
- Specific treatments
  Many interventions contribute to increase functional capacity, including:
  - General conditioning
  - Improving physical impairments (strength, balance)
  - Training to compensate for deficits (bowel training programs, cognitive retraining)
  - Incorporating medications as needed
  - Incorporating adaptive equipment
  - Learning new skills (vocational re-training)
  - Psychosocial adaptation
  - Continual practice with designated regimens
  - Motivational strategies
- Follow through: ongoing planning
  - Adherence to the program initiated
  - Reassessing and monitoring progress and progression, or identifying new issues
  - Continuous availability of the team and open communication
  - Interaction with community resources (MS organizations, visiting nurses)
  - Re-evaluation by the team in 3–12 months
  - Reasonable long-term program planning

## Rehabilitation team management of specific problems in MS

The rehabilitation team should be involved in the diagnosis and treatment of all problems in the MS patient. Each team member contributes to the successful management of the specific problem. Table B lists areas where each team member contributes their specific expertise. However, the entire team works together in complex areas such as fatigue, cognition, mood dysfunction, and pain, as well as bowel, bladder, and sexual problems. The interdisciplinary team communications is vital to the overall success. The common problems will be dealt with one by one.

### Fatigue

Fatigue is the most common symptom in MS and impacts the rehabilitation team's ability to improve both physical and cognitive activities.[25–27] Fatigue and cognitive dysfunction are major contributors to loss of employment. Fatigue may be secondary to the disease, medications, depression, heat, pain, infection, sleep deprivation, and stress.

The team approach to the treatment of fatigue begins with the consideration of the underlying issues. It must be dealt with by the entire team. The appropriate combination of exercise and adequate rest is added to the treatment of the specific underlying conditions contributing to fatigue. Fatigue is reduced by adjusting activity schedules so as to avoid overheating; by cooling techniques, e.g., cool baths, sucking on ice chips, and wearing cooling devices, such as a vest[28] can be combined with specific rehabilitation therapy programs. Monitoring fatigue with the Fatigue Impact Scale (FIS) and MSQOL-54 can help predict overall functioning.

Fatigue may be very variable and response to therapy can be complex. Although fatigue may be independent of depression and cognitive dysfunction, these three conditions often occur simultaneously and interact to increase the magnitude of the disability.

Rehabilitation works to diminish the various factors that contribute to fatigue. The specific approaches to nerve disturbance, pain, spasticity, sleep dysfunction, and medication evaluation etc. are combined with home and work environment modification and modifications of activities of daily living. Treating fatigue with an interdisciplinary approach not only improves the fatigue but also enhances the overall performance of MS patients.[29,30] A graded exercise program with time-to-rest periods and avoidance of overheating are advised.

In addition to the rehabilitation approach, medications may be indicated. A first-line therapy is amantadine hydrochloride, 100 mg twice a day.[31] Sometimes, an initial improvement is followed months later by a falloff in efficacy. Modafinil is often preferred by neurologists. Clinical trials of modafinil have shown mixed results but two studies have indicated good efficacy.[32,33] CNS stimulants such as methylphenidate have also been shown to improve fatigue and cognitive performance. Side effects include behavior disorders and sleep dysfunction, as well as the potential for habituation

### Weakness

‘Weakness is the most feared symptom of MS because it implies paralysis, loss of ability to walk, and severe disability’ said Petajan.[34] The rehabilitation team focuses on reducing the impact of weakness, preserving mobility, and enhancing quality of life.

Among the first questions that patients ask when given the diagnosis of MS are those related to their level of activity: What can they do? What activities should they refrain from? Should they stop all physical activities and just rest? Will exercise make them worse or will it keep them strong? Does MS affect the heart muscles?

Early in the disease, most MS patients have little or no problems with muscle function after a remission. Nonetheless, the rehabilitation team encourages them to exercise and physical therapists may supervise their exercise program. Since fatigue may be problematic, energy conservation techniques are developed by the occupational therapist and/or other team members. Early in their disease, MS patients usually have low disability scores and can use exercise to increase their aerobic capacity. As the disability increases, exercises are modified depending on the degree of weakness, spasticity, incoordination, and diminution in aerobic capacity. A reasonable exercise program can be prescribed by the physical therapist, regardless of the disability level. Maintaining cardiovascular fitness increases mobility and decreases fatigue. Exercise programs should be done three times a week if possible. The exercise program should be monitored, since excessive exercise may increase the body temperature and increase weakness and other MS symptoms.[28]

Overall muscle weakness, fatigue, and depression are improved through rehabilitation and exercise.[35] Exercise can encompass many activities and usually begins with a warm-up period to reduce strain and spasticity. Aerobic training is designed to improve fatigue, endurance, balance, flexibility, and strength. Muscle strengthening programs need planning and close supervision by a physical therapist to ensure maximum benefit and safety. Even patients in wheelchairs can usually benefit from a well-planned exercise program and reduce the risk of developing decubitus ulcers. Passive range-of-motion stretching exercise may be helpful in initial programs for those who are relatively more disabled. Progressive resistance exercises are more likely to be effective in patients with only mild disability.[39]

Schapiro developed a ‘maintenance rehabilitation’ concept with an ongoing rehabilitation program of physical and occupational therapy, which demonstrated superior efficacy, compared to a wait-listed control group, in outcomes such as ADLs, fatigue, pain, and general health measures on the SF-36 test.[37–39] Another study utilizing a comprehensive rehabilitation team approach demonstrated improvement in disability scores lasting up to 6 months.[40] These and other studies emphasize the importance of initiating an exercise program early in the course of MS and continuing the program with modifications even as disability increases.

Drug therapies in patients with weakness are usually designed to reduce spasticity and pain. These specific therapies are covered elsewhere in detailed by Schapiro.[6] Phase 3 clinical trials of 4-aminopyridine (or Fampridine SR) are encouraging and demonstrate that treatment can provide increased endurance and strength.

### Balance and gait

Improving balance and gait involves many issues such as ataxia, strength, vision, spasticity, tremors, and fatigue.[7] An interdisciplinary approach is important. Pharmacological agents have limited usefulness. Safety is a primary concern. Appropriate adaptive equipment can help maintain function as disability increases. However, MS patients may be reluctant to use assistive equipment and often need training and encouragement from the MS team, especially if safety is an issue. Using assistive devices can reduce fatigue and frustration, and patients will have the energy to accomplish more once they reach their destination.

Decreased balance contributes to mobility problems. Medications for balance problems are limited. However, decreasing fatigue and tremors and increasing endurance may help. Vestibular rehabilitation attempts to help patients adapt to balance problems. Some equipment has been designed to improve stability. Assistive devices, hand rails, and safety training can reduce the risk of falling.

Specific balance and gait interventions depend on the specific impairments that contribute to the problem. For example, maintaining postural control is an important outcome.[41] Programs such as Tai Chi,[42] Yoga, aquatics[43] and Feldenkrais[42] may be helpful in maintaining gait and balance function.

### Mobility and activities of daily living (ADL)

In a disabled patient, inputs from almost every member of the rehabilitation team are necessary to maximize function.[44] Maintaining adequate strength, balance, flexibility, and range of motion are complex issues. Contractures contribute greatly to reduced mobility. The physical therapist can supervise an exercise program. Stretching exercises are helpful as an initial step. Drugs to decrease spasticity are important, but the effects must be monitored because reduced spasticity might decrease gait performance in those patients who depend on a certain level of spasticity to walk. Specifically, some patients ‘walk on their spasticity,’ with the stiff leg providing support for ambulation. Judicious use of botulinum toxin injections can be very helpful in selected MS patients to reduce spasticity.

Assistive technology can increase the level of function. Ankle-foot orthoses are light plastic devices that can improve gait by ensuring better foot dorsiflexion. Canes with a single point or four points may be useful, depending on the disability. Forearm crutches may help those with weakness. Walkers help both balance problems and weakness, while providing more safety than canes or crutches.

The use of wheelchairs and scooters is often misunderstood. Too often patients do not have access to these early enough in their disease course. For example, some MS patients have the strength and balance to walk with a walker but are exhausted after a short walk. Therefore, they cannot function when reaching their destination. Many patients use scooters that are not properly fitted, which can create additional problems. The timing of the utilization of a manual wheelchair *vs* a motorized wheelchair is a decision that should be left to the appropriate rehabilitation therapist and the patient. Not all wheelchairs are alike; they can serve different purposes. A poorly fitted wheelchair can cause increased pain, spasticity, decubitus ulcers, and safety problems. Proper wheelchair seating needs an individualized approach to assure appropriate thoracolumbar, truncal, and pelvic support.

The occupational therapist and/or the rehabilitation nurse can help patients improve their activities of daily living as well as their mobility. Practical techniques and assistive devices ease the burdens of dressing, bathing, eating, household chores, and daily care.

### Cognitive dysfunction

In spite of early descriptions of cognitive dysfunction in MS, before the 1980s MS was thought to only rarely affect cognition. Now cognitive dysfunction is recognized as an important factor affecting the patient's vocational opportunities, social adaptability, and quality of life.[45] The prevalence of cognitive dysfunctions is estimated to be as high as 70% and most patients with cognitive dysfunction are unemployed within 10 years of disease onset. Cognitive dysfunction may occur very early in the disease and be independent of physical disability.[46]

Cognitive dysfunction may not be recognized by the healthcare professional or even the patient. Therefore, the first step to cognitive rehabilitation and re-training is recognition. The bedside mini-mental status examination and other brief clinical tests underestimate the true incidence of cognitive dysfunction.[47] While a formal neuropsychological evaluation is the most accurate diagnostic tool, the testing may take several hours and can be exhausting as well as expensive. IQ testing may not fully disclose impairments. Tests of learning, memory, attention, concentration, abstract reasoning, problem solving, executive dysfunction, working memory, visuospatial, and constructional tasks such as following maps and performing complex assemblies often reveal cognitive dysfunction.[48] Neuropsychological evaluations are problematic because of the paucity of expertise, the length and expense of much of the testing procedures, the lack of financial reimbursement in some situations, and the lack of follow-through cognitive retraining for the patients.

Cognitive dysfunction in MS is complex since it may be associated with continuing disease activity, acute exacerbations, fatigue, depression, medications, sleep disorders, and general medical conditions such as anemia, thyroid dysfunction, and infections.

The management of cognitive dysfunction is difficult. Alzheimer's disease medications have had limited success.[49] Cognitive retraining therapy is an interdisciplinary rehabilitation approach that can help individuals adapt and cope with their cognitive dysfunction. Compensatory strategies such as appropriate time management, improved sleep hygiene, medication adjustments, memory queues, and calendars are often helpful. Modafinil (Provigil^®^) and CNS stimulants may improve attention span, working memory, and phonemic fluency.[32] Immunomodulating therapies have been shown to improve cognition in some clinical trials.[50–52]

### Pain

Charcot described pain in MS in 1872, but the association of significant pain was not well delineated until recently. Now pain syndromes are recognized as significantly impairing quality of life in 45–65% of MS patients.[53,54] Pain can occur early in the disease and may be associated with the disease process directly (central neurogenic pain) or may occur as a consequence of MS symptoms such as weakness and coordination. Trigeminal neuralgia in young individuals is highly suggestive of MS.[55]

Burning or dysesthetic pain may be associated with a hyperpathic quality with an increased sensitivity to tactile stimulation. Paroxysmal pain can be quite distressing.[56] Lhermitte sign involves an electric shock sensation radiating from the neck down when the neck is flexed. The neurogenic pain will often respond to antiepileptic drugs as gabapentin, pregabalin, or carbamazepine. Baclofen may also be helpful. Refractory pain may respond to neurosurgical procedures. Axial skeletal pain, such as back pain, is often seen in MS patients with an abnormal gait, disequilibrium, or muscle activities created by distortion of the axial skeletal system. This type of pain may be treated with a variety of rehabilitation techniques to strengthen the skeletal muscle system and improve posture, truncal stability, and balance. Typical radicular pain can be experienced in an exacerbation of MS. Treatment with steroids is helpful.[57]

### Mood disorders

Mood disorders occur in over 50% of MS patients.[58] Depression is the most common mood disorder. Depression may be related directly to the disease or may be an associated factor related to situational factors. The risk of suicide in MS patients is estimated to be seven times higher than in the general population.[59] Depression is a major contributor to the decline in quality of life in MS patients. The problem is confounded by the fact that it is often underdiagnosed and undertreated in MS.

Early recognition and treatment of mood disorders are imperative. The rehabilitation team may discover the initial symptoms of depression. Depression may herald an impending exacerbation or change in the disease course. Self-reported instruments for measurement of mood disorders such as the Beck Depression Inventory may be useful for screening individuals at risk.[60]

The treatment of depression in MS usually combines medication and psychotherapy. A supportive rehabilitation team is necessary. Medications used in depression are covered in the accompanying article on symptom management.[7] Antidepressant drugs may take weeks to months to reach maximum effectiveness. The side effects of these drugs may compound other MS symptoms such as sexual dysfunction. The combination of psychotherapy and antidepressants has been shown to be better than antidepressants alone.[61] Caregiver support is an essential part of the psychotherapy process. However, depression and anxiety in caregivers is common, which increases the stress on the MS patient and vice versa. The rehabilitation team can help identify contributing factors and monitor the progress of therapy.

Rapid mood swings resulting in emotional lability and rapid oscillations of mood from euphoria to inappropriate crying are at times noted (pseudobulbar affect). This problem can often affect social functioning and quality of life for both patients and their families. Education, psychotherapy, family counseling, and tricyclic antidepressants can all help as the patient and caregiver struggle to understand the condition and its consequences.

Medication used to treat MS symptoms and exacerbations may produce hypomanic states, which may be difficult to separate from the direct effects of the disease. Steroid therapy is an example. Education by the rehabilitation team and sedative medications may improve the tolerability of steroids. Interferons may increase the risk of depression. Close monitoring and early intervention often reduce the need to discontinue these treatments.

### Bowel dysfunction

Bowel dysfunction occurs in over 50% of patients with MS. It is usually associated with bladder dysfunction.[62,63] The rehabilitation team should be adept at analyzing the multitude of issues contributing to this problem. The rehabilitation nurse often leads these efforts, while other members of the team add valuable input. For example, the physical therapist can help with an exercise program and the nutritionist can modify the diet. Special attention to hydration, exercise, bowel habits, diet, and medication side effects are important.

The most common gastrointestinal symptoms include delayed gastric emptying, constipation, and fecal incontinence. Constipation may result from direct neurologic factors such as delayed gastrointestinal transit time or weakened abdominal muscles, which inhibit effective bowel evacuation. Indirect factors such as fluid restriction, poor dietary habits, inconsistent bowel programs, and medications may also affect constipation. Medications associated with constipation include antacids, anticholinergic medications (given for bladder control), tricyclic antidepressants, some antihypertensives, narcotics, nonsteroidal anti-inflammatory medications, and muscle relaxants.

Fecal urgency and incontinence is secondary to interruption in sacral neural pathways, which may impair critical awareness of defecation. Impaired functioning of the anal sphincters and rectal muscles may also result in sudden defecation, with or without urgency.

Assessment of bowel function should be a routine part of the history and examination. The goal of therapy is to establish a regular bowel pattern for defecation, normalize stool consistency, and stimulate bowel emptying on a regular basis to avoid both fecal impaction and incontinence.

Managing fluid and fiber intake, as well as encouraging regular exercise, are important interventions. At least 2 l of fluids daily are recommended. An increase in fiber intake results in softer and bulkier stools, which improves transit time and may help both constipation and incontinence. High-fiber foods such as cereals, fruits, and vegetables are encouraged. Fiber supplements are available and can be used.

A bowel program to establish a regular pattern of elimination is often an early step in bowel management. The urge to have a bowel movement is strongest 20–30 min after consuming a meal. Therefore, patients are encouraged to time their attempts to defecate following meals. If a bowel program is not completely effective, medications are available. Stool softeners such as dioctyl sodium sulfosuccinate or Colace^®^ may decrease the hardness of stools by drawing water into the bowel. Bulk-forming laxatives such as Metamucil^®^ or Citrucel^®^ may be taken 6–8 h before expected elimination. Oral stimulants act by stimulating parasympathetic reflexes to produce peristalsis; they include Senna^®^, Ex-Lax^®^, Dulcolax^®^, Milk of Magnesia^®^, or Peri-Colace^®^. These stimulants may be irritant to the rectum and may also be habit forming.

If these measures fail, rectal suppositories may be used in conjunction with other agents to promote elimination. Glycerin suppositories produce local stimulation and a lubricant for a passage of stool. Dulcolax^®^ suppositories induce strong involuntary contractions that facilitate elimination of stool. Suppositories usually work within 15 min to 1 h but may not be as effective if stool is present in the rectum. Enemas may be used when other measures fail. Enemas may result in dependency if used regularly. The patient should first be evaluated for fecal impaction and if this is present digital evacuation may be necessary. The presence of fecal impaction indicates the need for an aggressive bowel program.

Patients with fecal incontinence should be advised to attempt elimination 20–30 min after consuming a warm meal or beverage, most often in the morning. Patients are advised to sit on the toilet for 10 min and try to bear down. Rocking back and forth on the toilet and massaging the abdomen may promote bowel activity. The use of suppositories to stimulate rectal emptying may be necessary. Adequate fluid and fiber intake is necessary for maintaining stool consistency.

It may take several weeks for these interventions to be effective. The patient should understand that the goal of the bowel program is to have predictable regular eliminations with a soft, formed stool, which decreases the problems of constipation or fecal incontinence. Another goal of an adequate bowel training program is long-term avoidance of strong laxatives and enemas.

### Bladder dysfunction

The neurologic control and management of bladder function is a very complex and hierarchal process. This includes cerebrocortical centers, thalamic and midbrain regions, as well as brainstem and spinal cord components. MS lesions and associated disturbances within the CNS can impair this sophisticated control, leading to retention, urgency, incontinence, and other associated problems.

The primary neurologic systems involved include the parasympathetic control of the detrusor muscle of the bladder, sympathetic innervation of the internal urethral sphincter, and somatic nerve supply to the external sphincter and other striated muscles. In the resting state, the detrusor muscle is tonically inhibited via the parasympathetic nervous system, while the sympathetic nervous system tonically contracts the internal sphincter. This allows urine to be collected and stored within the bladder, without leakage or incontinence.

Micturition, or bladder voiding/emptying, is triggered by relaxation of the internal sphincter. This is followed by diffuse contractions within the detrusor muscle leading to emptying of urine through the urethra. This may be complemented/augmented by increasing abdominal pressure through voluntary contraction of the abdominal skeletal muscles (as is done during the Valsalva maneuver). Normally, only a few milliliters (ml) of urine remain in the bladder after completion of this process.

### Hyperactive bladder

Over time, many patients with MS and a neurogenic bladder develop hyperactivity of the detrusor muscle, which causes bladder irritation and frequent voiding of small amounts of urine. Oftentimes this is accompanied by inadequate relaxation or dyssynergia of the internal sphincter. Such patients may benefit from a trial of anticholinergic medications such as oxybutynin (Ditropan^®^) or tolterodine (Detrol^®^) that can dampen/inhibit detrusor firing activity. In such patients, low doses of oxybutynin or tolterodine is initiated at bedtime to decrease nocturia. If feasible, post-void residual (PVR) urine levels are monitored. The goal of therapy is to have less than 100 ml of PVR urine and a continent patient.

If bladder urgency and/or incontinence during the daytime persist, the patient is advised to take the anticholinergic medication twice daily. Monitoring bladder function with PVR urine measurement may be necessary. The adverse effects of these anticholinergic drugs (e.g., impairment of cognition, constipation, dry mouth, and fluid retention) may mimic MS symptoms.

During the bladder training program, it may be necessary to limit fluid intake to less than 2000 ml a day, depending on the patient's response. Increasing fluids during the daytime and significantly decreasing the fluid intake in the evening and at night is prudent. The body tends to make more urine at night when the patient is in a recumbent position. Desmopressin (DDAVP) at bedtime may be helpful to reduce nocturia.

### Hypotonic bladder

Urinary retention due to a hypotonic bladder is less frequent and is more difficult to manage. In a severe MS exacerbation, bladder hypofunction may occur acutely, with decreased vitality of detrusor muscle contractions and a concomitant incomplete relaxation of the internal sphincter. This urinary retention leads to overdistention of the bladder, overflow incontinence, and sometimes reflux of urine up the ureters toward the kidneys. Bladder ultrasound can be used to monitor bladder volume. Alternatively, intermittent catheterization can also document bladder volume. Normally, bladder micturition or voiding reflexes are triggered by urine volumes between 200 and 400 ml. In MS, this threshold may be increased.

An indwelling (Foley) catheter may be temporarily necessary during an MS exacerbation. When the catheter is removed, the bladder training program begins with the process of timed voiding. The patient is instructed to sit on the toilet about every 3 h and to attempt to void. After every other voiding attempt (or about four times a day) PVR urine is measured by ultrasound or intermittent catheterization. The target/goal of the bladder program is for the patient to successfully void and be continent with PVR urine volumes less than 100 ml. If PVR urine volumes exceed 100 ml, medication with bethanechol (Urecholine^®^) to increase detrusor contractions may be tried. While the effects may be disappointing, this is the only oral agent available to augment detrusor contraction. Also, decreasing internal urethral sphincter tone with agents such as tamsulosin HCl (Flomax^®^) or terazosin (Hytrin^®^) may be helpful. Monitoring for orthostatic hypotension about 2 h after administration of these drugs is recommended.

If the bladder volume continues to exceed 300–400 ml, intermittent catheterization can be helpful. The normal maximum volume of the bladder is 400–500 ml. Greater volumes will adversely affect the vitality of detrusor muscle contractions and also increase the risk of reflux of urine to the kidneys, which has the potential to cause serious kidney infections and other renal problems.

If adequate bladder emptying cannot be accomplished, the staff should proceed with training the patient and family/caregivers in intermittent catheterization techniques. If satisfactory management is still not achieved, as a last resort, a chronic indwelling catheter is an option. Sometimes, with an indwelling catheter, medication such as oxybutynin (Ditropan^®^) or tolterodine (Detrol^®^) to dampen detrusor contractions may be helpful. These medications help lessen the risk of reflux of urine to the kidney.

Male patients with chronic indwelling Foley catheters are at risk for potential serious complications such as refractory urethritis, prostatitis, urethral strictures, and even traction necrosis of the penis. Because of these risks, male patients may benefit from a suprapubic catheter placement. Female patients with chronic indwelling Foley catheters require suprapubic catheter placement less frequently due to the shorter length of their urethras.

Urine is colonized/contaminated with bacteria when a chronic indwelling catheter is used. However, antibiotic treatment is usually reserved for patients with signs and symptoms of a urinary tract infection. Continual and/or repeated use of bactericidal agents will increase the risks for colonization with resistant organisms over time. Low-dose administration of a bacteriostatic medication and acidification of urine are options.

### Sexual dysfunction

Sexual dysfunction is common in both males and females with MS due to the physical, cognitive, and emotional manifestations of the disease. Some degree of sexual dysfunction is encountered in most males and females, ranging up to 91% in some surveys.[64–67] In addition to the physical impact, the ability for individuals to form intimate relationships may be affected. The Multiple Sclerosis Intimacy Questionnaire (MSISQ) is a 19-item questionnaire developed to measure sexual dysfunction. This tool allows the clinician to pinpoint areas of sexual dysfunction and their severity.

Sexual dysfunction may be a direct reflection of MS lesions manifested by neurologic impairments, including erectile dysfunction, impaired genital sensation, and decrease vaginal lubrication. However, other MS symptoms may produce indirect changes in sexual responsiveness. These symptoms include fatigue, spasticity, spasms, bowel and bladder dysfunction, mobility difficulties, muscle weakness, contractures, indwelling catheters, incontinence, and side effects from medications.[68] Psychological issues such as depression, anxiety, and anger may impact sexual function and intimacy in relationships. Other issues associated with MS, such as loss of employment, may lead to negative self image and changing roles in relationships. The rehabilitation team works together to deal with these multiple issues.

Managing sexual dysfunction begins with educating the patient and the partner. Redefining sexual intimacy and promoting better communications around sexual issues are keys to success. Neurologists, gynecologists, and clinical psychologists may articulate the various issues and help the rehabilitation team in improving the patient's sexual function, relationships, and overall quality of life. Lifestyle issues such as alcohol, drug abuse, depression, and cognitive issues are important parts of the evaluation. Medications that may help in sexual dysfunction include antidepressants, anticholinergics, anticonvulsants, and antihypertensive mediations; alternative medicine is also reported to be of use.

Specific symptoms such erectile dysfunction may require phosphodiesterase inhibitors such as sildenafil (Viagra^®^), tadalafil (Cialis^®^), or vardenafil (Levitra^®^). Penile injections with prostaglandin E1 or papaverine may be effective. Vacuum suction devices and surgical implantation of inflated or noinflated devices may also be an option.

One of the most common problems for women is lack of vaginal lubrication. Soluble water lubricants such as KY Jelly^®^ are often helpful. Education about alternative ways to achieve physical sexual satisfaction (e.g., variation of positions or foreplay techniques) is often helpful.

Rehabilitation therapists and nurses work together to address the many medical and psychological issues related to self-esteem, such as negatively altered body image and diminished sexual confidence. Counseling is important for the both the patient and the partner. When available, couples-oriented sexual therapy practitioners can often improve the sexual experience for patients and their partners by enhancing communication skills and improving the overall quality of the relationship. In spite of physical limitations, maintaining intimacy in the relationship is possible with counseling and education. Good communication is a key ingredient.

### Dysarthria and Dysphasia

Dysarthria and dysphasia may or may not occur concurrently.[69] The speech and language pathologist specializes in evaluating and treating speech, communication, cognitive, and swallowing problems. Dysphasia evaluation may include videofluoroscopic or barium swallow studies to track the movement of food during swallowing.[70,71] Treatment is rehabilitative and educational. Changing the head or body position during eating may relieve the symptom. Eating smaller quantities at any one time may reduce fatigue and choking.

## Conclusion

Interdisciplinary team rehabilitation is an integral component of the continual care for the MS patient. Numerous studies have documented the effectiveness of rehabilitation, which address impairments, disabilities, and handicaps. Early in the disease course, in addition to specific MS therapies, the focus includes adapting to changes brought on by MS within the patients' body, their families, their vocations and advocational pursuits, and their psychological state. A proactive approach to health, education and maintenance, exercise and energy conservation, employment, and relationships will minimize the impact of future symptoms.

Later in the disease course, rehabilitation is designed to prevent complications and minimize functional deterioration; specific problems that are addressed are weakness, spasticity, mobility, cognition, mood, pain, speech and swallowing, as well as bladder, bowel and sexual dysfunction. The rehabilitation team works with patients and family on both physical and psychosocial issues to promote a higher quality of life. Quality of life depends on maximizing one's health, maintaining good relationships, feeling productive, and pursuing creative endeavors. The health care team plays an important role in all of these pursuits.
